# The Dilemma of the Little Hospitals

**Published:** 1920-08-07

**Authors:** 


### THE MATRONS' AND SISTERS' DEPARTMENT.

### THE DILEMMA OF THE LITTLE HOSPITALS.

Every onward step towards organisation plunges the little institutions deeper into difficulties. Time was when a nurse could qualify without misgivings in hospitals containing less than forty beds, and take her place without question in the nursing world. When there came to be trained matrons from good schools in these establishments who taught, and trained, and carried on the traditions which they themselves had learned, the little hospitals turned out many women who, in all but a wide experience of medical and surgical work, might be reckoned good nurses. These days have gone by. The probationer now enquires anxiously whether the certificates which the institution offers is "recognised," or before she is allowed to sign on for a term of years the matron herself informs her that she must regard her term of service as merely preliminary to a term of real training. Under these circumstances it is getting increasingly difficult to attract probationers at all.

We are told that the remedy for all the disabilities under which small and special hospitals labour is to be found in linking them up with the larger and general ones. It is quite true that by a judicious system of linking something can be done to relieve the plight of the institutions which give a one-sided training. Women's hospitals can be linked with men's. Fever and consumption hospitals can be linked with general hospitals, and already some excellent examples of co-operation have been effected in this direction. It is probable that this system, under the auspices of the General Nursing Council, may see a wide expansion. And yet it is pretty certain that, whatever may be done in this direction, certain establishments will be left outside as not offering training which could under any circumstances be allowed to count. Cottage hospitals containing less than a prescribed number of beds cannot be allowed to give training. Small special hospitals are even less admissible. What is the remedy?

In our judgment it is too much to ask of young women adopting the nursing profession to spend a couple of years in an institution which cannot advance them on their way. There may be special circumstances, such as the timidity of the probationer or her parents' doubts about her health or vocation, which make it convenient for a candidate to begin work in a small hospital, and there are many valuable nurses now in the profession who would perhaps never have achieved their training if they had not begun it under sheltered conditions, which gave them confidence to advance later on to the real thing. But these cases are exceptional. It is impossible to staff the little hospitals on exceptions, and the pressing need is to find probationers for them.

Would it not be wiser to recognise that these hospitals, though well adapted to give short periods of preliminary instruction in nursing work, are not suited to the requirements of the professional nurse? Women who do not intend to take on the career of nursing are shy of binding themselves for a long-term period. There is a very large body of women, however, who crave for such an introduction to nursing as would help to make them useful in their own homes and as Red Cross workers. The remedy of the little hospitals is to get into touch with these often enthusiastic people. At present, ambitious training schemes stand in the way. After one year the probationer has learned all that she is likely to learn, and the next step is to grow stale. It is probable that matrons could get as many helpers as they want on a basis of six months to a year's residence. For the most part the duties of these probationers would be of a routine nature, though a friendly matron would give them the opportunity for which they crave of learning the elements of nursing. War conditions opened out once for all the possibilities for service in hospital possessed by complete novices, and a succession of eager nursing pupils received as a favour from the local Red Cross organisation would prove far more helpful, it may well be believed, than the bored probationers who have repented them too late of entering an institution of which the certificate does not "count."

The little special hospitals can generally, we believe, attract staff-nurses anxious to obtain experience in their particular branch of work. But here again an injudicious term is often laid down. It is a term of intensive training which is sought, not a long period of service with a few lectures tacked on. All the nurses who add special work to their training are in a hurry to be doing something else. Their needs must be studied, their point of view respected. Too long the question of qualifying has been regarded from the hospital standpoint. It is necessary to study also that of the nurse herself.

